# Role of Isolated Limb Perfusion in the Era of Targeted Therapies and Immunotherapy in Melanoma. A Systematic Review of The Literature

**DOI:** 10.3390/cancers13215485

**Published:** 2021-10-31

**Authors:** Lourdes Sevilla-Ortega, Lara Ferrándiz-Pulido, Natalia Palazón-Carrión, María del Carmen Álamo de la Gala, Rubén de Toro-Salas, José Garnacho-Montero, José Antonio Marcos-Rodríguez, Ana Agudo Martínez, Omar Araji-Tiliani, María Cinta Calvo-Morón, José Miguel Barquero-Aroca, Antonio Ramón Fernández-López, José María Jaime-Borrego, Juan Carlos Santos-Jiménez, David Moreno-Ramírez, Luis de la Cruz-Merino

**Affiliations:** 1 Clinical Oncology Department, Universitary Hospital Virgen Macarena, 41003 Seville, Spain; lourdes.sevilla.sspa@juntadeandalucia.es (L.S.-O.); natalia.palazon@juntadeandalucia.es (N.P.-C.); mcarmen.alamo.sspa@juntadeandalucia.es (M.d.C.Á.d.l.G.); ruben.toro.sspa@juntadeandalucia.es (R.d.T.-S.); 2 Dermatology Department, Universitary Hospital Virgen Macarena, 41003 Seville, Spain; lferrandiz@e-derma.org; 3 Intensive Care Department, Universitary Hospital Virgen Macarena, 41003 Seville, Spain; jose.garnacho.sspa@juntadeandalucia.es; 4 Pharmacy Department, Universitary Hospital Virgen Macarena, 41003 Seville, Spain; jose.marcos.sspa@juntadeandalucia.es; 5 Radiopharmacy Department, Universitary Hospital Virgen Macarena, 41003 Seville, Spain; ana.agudo.sspa@juntadeandalucia.es; 6 Cardiovascular Surgery Department, Heart Area, Universitary Hospital Virgen Macarena, 41003 Seville, Spain; omar.araji.sspa@juntadeandalucia.es (O.A.-T.); josem.barquero.sspa@juntadeandalucia.es (J.M.B.-A.); chusco6465@gmail.com (J.M.J.-B.); jucasaji1969@gmail.com (J.C.S.-J.); 7 Nuclear Medicine Department, Universitary Hospital Virgen Macarena, 41003 Seville, Spain; mariac.calvo.sspa@juntadeandalucia.es; 8 Anesthesiology and Reanimation Department, Universitary Hospital Virgen Macarena, 41003 Seville, Spain; antonior.fernandez.sspa@juntadeandalucia.es; 9 Medicine Department, Universidad de Sevilla, 41003 Seville, Spain

**Keywords:** malignant melanoma, chemotherapy, isolated limb perfusion, melphalan, tumor necrosis factor

## Abstract

**Simple Summary:**

Melanoma still represents a major challenge for health systems around the world. A classical treatment for patients with a high tumor burden or rapidly recurrent in-transit metastases is isolated limb perfusion (ILP) therapy instead of locoregional surgical resection, or when the latter is no longer feasible. In this era of modern systemic treatments for melanoma, it still remains interesting to analyze the role of management approaches for locoregionally-advanced disease, such as isolated limb perfusion (ILP). With this purpose, we conducted a systematic review updating the available literature on ILP for malignant melanomas. The main objectives of this review were to focus on the effectiveness and safety of ILP. In conclusion, ILP, with its low incidence of regional and systemic toxicity, is a valuable palliative treatment not only for patients with disease confined to a limb, but also for patients with a metastatic melanoma with a bulky or symptomatic disease, in order to improve their quality of life.

**Abstract:**

Background. Isolated limb perfusion (ILP) is a locoregional procedure indicated by the unresectable melanoma of the limbs. Its complexity and highly demanding multidisciplinary approach means that it is a technique only implemented in a few referral centers around the globe. This report aims to examine its potential role in the era of targeted therapies and immunotherapy by conducting a systematic review of the literature on ILP. Methods. PubMed, Embase and Cochrane Library were searched. The eligibility criteria included publications from 2000–2020 providing valid data o effectiveness, survival or toxicity. Studies in which the perfusion methodology was not clearly described, letters to the editor, non-systematic reviews and studies that applied outdated clinical guidelines were excluded. To rule out studies of a low methodological quality and assess the risk of bias, the following aspects were also required: a detailed description of the applied ILP regimen, the clinical context, follow-up periods, analyzed clinical endpoints, and the number of analyzed ILPs. The disagreements were resolved by consensus. The results are presented in tables and figures. Results. Twenty-seven studies including 2637 ILPs were selected. The median overall response rate was 85%, with a median complete response rate of 58.5%. The median overall survival was 38 months, with a 5-year overall survival of 35%. The toxicity was generally mild according to Wieberdink toxicity criteria. Discussion. ILP still offer a high efficacy in selected patients. The main limitation of our review is the heterogeneity and age of most of the articles, as well as the absence of clinical trials comparing ILP with other procedures, making it difficult to transfer its results to the current era. Conclusions. ILP is still an effective and safe procedure for selected patients with unresectable melanoma of the limbs. In the era of targeted therapies and immunotherapy, ILP remains an acceptable and reasonable palliative treatment alternative, especially to avoid limb amputations. The ongoing clinical trials combining systemic therapies and ILP will provide more valuable information in the future to clarify the potential synergism of both strategies.

## 1. Introduction

Melanoma still represents a major challenge for health systems around the world, due to its rising incidence and the certainty that an early detection would mean a cure for most of the patients. Nevertheless, in the advanced stages and especially when some risk factors are present (node involvement, ulceration), melanoma cases imply a greater complexity which causes a high morbi-mortality. As stated, melanoma incidence continues to increase in the Western world: according to GLOBOCAN 2020 [1], the expected world number of new cases of CM was 324,635 in 2020, with an age-standardized incidence rate of 3.2 per 100,000/year and a mortality rate of 0.57 per 100,000/year.

Although most of the patients are diagnosed in early stages of the disease, approximately 5–8% of patients with melanoma recurrences will develop in-transit metastases, that is, multiple recurrent tumor deposits in the superficial lymphatic vessels, most often confined to the extremities [2]. Among these patients, the quality of life is greatly compromised, mainly due to tumor burden-related complications [3].

A classical option for patients with a high tumor burden or rapidly recurrent in-transit metastases is isolated limb perfusion (ILP) therapy instead of a locoregional surgical resection, or when the latter is no longer feasible. This technique was first unveiled in 1958 by Creech and Krementz [4]. The procedure consists of isolating the involved limb from the systemic circulation (using a properly placed pneumatic tourniquet or Esmarch bandage) and administering chemotherapy agents through a cannulated artery and vein using an extracorporeal bypass circuit, which allows the administration of a dose of cytostatics up to 20 times greater than the systemic dose [5]. In 1959, the first ILP intervention to treat a patient with an in-transit melanoma on one leg using melphalan took place (L-phenyl alanine mustard) [6]. In 1969, Stehlin combined moderate hyperthermia (40–41 °C) and ILP to enhance the effect of melphalan [7].

The tumor necrosis factor (TNF-α) is a cytokine with direct and indirect antitumor effects. Its effects may be mediated by a specific destructive effect against the tumor vasculature that is synergized with the cytotoxic effect of melphalan [8]. Since TNF-α is a key physiological mediator of the systemic inflammatory response, the systemic administration in doses with an antitumor effect has severe and potentially fatal side effects. Therefore, it can only be used clinically in ILP, and the continuous monitoring of the perfusion circuit leaks is an absolute requirement [9]. In 1994, Lienard et al. reported an additional positive effect with TNF-α and melphalan (TM-ILP) [10]. Subsequently, it is suggested on the basis of several case series that melphalan plus TNF-α ILP has higher overall and complete response rates than melphalan alone [11,12,13]. Despite this, phase III, randomized trials are lacking to fully elucidate the real value of ILP with or without TNF-α, and its eventual impact on survival over other strategies. Furthermore, the advent and development of immunotherapy and targeted therapies has dramatically changed the therapeutic landscape of patients with advanced melanoma, with a significant improvement in patient survival in the last decade. Therefore, in this era of modern systemic treatments for melanoma, it still remains interesting to analyze the role of management approaches for locoregionally advanced disease, such as isolated limb perfusion (ILP). With this purpose, we conducted a systematic review updating the available literature on ILP for malignant melanoma (MM). The main objectives of this review focused on the effectiveness and safety of ILP.

In this systematic review of the literature, we present 25 studies published between 2000 and 2019 that include a total of 2637 ILPs.

## 2. Materials and Methods

Between June and July 2021, searches in PUBMED, MEDLINE, and EMBASE were performed using the following list of keywords (intra-arterial chemotherapy, intra-arterial perfusion, isolated limb perfusion, cutaneous melanoma, malignant melanoma, transit metastases, satellite, locoregional metastases, melphalan, interferon alpha, tumor necrosis factor alpha, normothermia, hyperthermia, complete response, partial response, overall response, survival, overall survival, disease-free survival, toxicity, regional toxicity, systemic toxicity). We also searched the reference lists of previous systematic reviews, as well as the Cochrane database, where no studies were found.

To limit biases, the reviewers performed an exhaustive search of all relevant articles, explicit and reproducible selection criteria, assessment of the design and characteristics of the studies. The assessment of the methodological quality of the studies was carried out by five reviewers independently to avoid evaluation biases. We understand that one of the main biases of the review may be publication bias, since unpublished articles in the aforementioned databases were not taken into account.

Data analysis was performed using the SPSS version V28 program. The presentation of the tabulations was carried out using the Windows Excel and Word programs.

PRISMA statement has been followed to carry out this review and the Registration Number is reviewregistry1244.

### 2.1. Inclusion and Exclusion Criteria

Eligible studies had to meet the following inclusion criteria: (1) studies published between 2000 and 2020; (2) studies including subjects with unresectable MM of the extremities treated with any ILP regimen, regardless of temperature level (hyperthermia, normothermia) or the chemotherapy drug administered (melphalan, melphalan and TNF, others); (3) studies that analyze efficacy or effectiveness endpoints (clinical response, survival, recurrence rate, limb recovery rate); (4) studies that analyze safety parameters in terms of regional toxicity and/or systemic toxicity; (5) and Eligible study designs: randomized clinical trials (RCTs), cohort studies, case–control studies, and case series.

**Figure 1:**
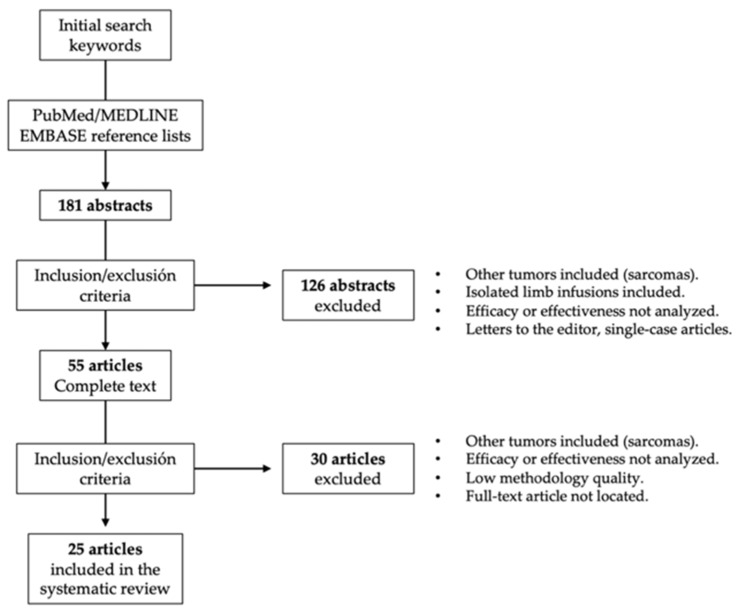
Procedure for the selection of studies included in this systematic review.

Studies in which the perfusion methodology (chemotherapeutic drug, temperature regimen, etc.) was not clearly described, studies that did not report valid results on clinical effectiveness or toxicity, letters to the editor, non-systematic reviews, and studies that applied outdated clinical guidelines were excluded from this systematic review (Figure 1). To rule out studies of low methodological quality, the following aspects were also required: detailed description of the applied ILP regimen, clinical context, follow-up periods, analyzed clinical endpoints, and number of analyzed ILPs.

Abstracts for which we could not located a full-text article were excluded, as were publications in a language other than English. Five reviewers collected the data independently by tabulating the study intervention characteristics and comparing them against the planned groups for each synthesis. Disagreements were resolved by consensus.

### 2.2. Outcome Measures

The RECIST and WHO criteria to assess tumor response to nonsurgical treatments were applied to extract data on objective clinical responses to ILP [14,15]. Therefore, the percentage of patients who achieved complete response (CR), partial response (PR), and overall response (OR) were the efficacy endpoints analyzed. Studies that did not provide direct information on these measures were also included if they could be calculated from the available data. In that regard, OR was calculated as the sum of CR and PR. The measure used to synthesize these results was the median, providing the interquartile range in all instances.

Survival after ILP was also analyzed. At this point, data on overall survival were extracted in terms of percentage (3-year and 5-year overall survival) and median overall survival. Median progression-free survival was also registered. Other secondary endpoints drawn from the analyzed studies were time to local progression (TTLP), time to systemic progression (TTSP), melanoma specific survival and the rate of limb recovery.

For the assessment of regional toxicity, studies describing the results according to the Wieberdink classification system for regional toxicity were included in the review [16]. For the analysis of systemic toxicity, the Common Terminology Criteria for Adverse Events version 3.0 [17], version 4.0 [18], version 5.0 [19] and the WHO classification of chemotherapy toxicity [20] were contemplated.

## 3. Results

Twenty-five studies were included in this review, representing a total of 2637 ILPs (Table 1), with a median of 91 ILPs included (range 17–380). Most were observational (88%, *n* = 22), while there were only three clinical trials [21,22,23] (12%), and only one of them was a randomized clinical trial comparing two chemotherapy regimens [21]. Four studies (16%) reported results on repeated ILPs [24,25,26,27]. All studies provided efficacy data in terms of the clinical response and toxicity except the study by Alexander et al. [28], which only reported efficacy data. The mean age of the patients was 64.07 years.

### 3.1. Clinical Response

All studies provided data on the clinical response. A median OR of 85.00% (range 55.00–100.00) was reported, with a median CR of 58.5% (range 0.00–89.20%) (Table 2).

The valid data on ILP efficacy with melphalan alone could be ascertained from seven studies that included 508 ILPs. They reported a median OR of 84.5% (range 79–90.6%), with a median CR of 57% (range 33–64%). Regarding the melphalan and TNF combination, twelve studies [21,22,24,26,27,31,34,35,37,41,42,43] (*n* = 855) reported valid data, with a median OR of 93.00% (range 67–100%) and a median CR of 61.5% (range 33–76%).

According to Rossi et al. [37], the complete response rate was higher among the patients who underwent isolated limb perfusion with TNF-α, with respect to those who had undergone isolated limb perfusion with only melphalan (60.3% versus 41.5%; *p* = 0.036). However, the aforementioned study failed to demonstrate significant differences between the melphalan monotherapy and the combination in the short-term response rate, with a complete response rate of 25% (14 of 58 patients) in the melphalan arm and 26% (15 of 58 patients) in the melphalan-plus-TNF-α arm (*p* = 0.435 and *p* = 0.890, respectively).

In general, in the studies with a greater number of patients included [21,27,33,34,35,37,41,43,45]. Nevertheless, other therapies were also included in the literature. Specifically, two studies included the combination of melphalan with D-actinomycin (*n* = 243; OR 87.65% [range 80.70–96.60%]; CR 76% [range 62.8–89.20%]); and one included the combination of melphalan with dacarbazine (*n* = 100; OR 90.80%; CR 73.40%). In addition, a pilot trial [23] that included 17 patients used melphalan combined with L-19 TNF at different doses (325 µg and 650 µg) reported CR in half of the patients (5/10) who received the 650 µg dose, since none of the seven patients in the 325 µg dose cohort achieved this. Rossi et al. [22] compared the combination of melphalan, TNF-α and IFN α-2b versus melphalan and TNF-α. 50% responses (12/24) were observed in the first group and 53% (10/19) in the second group. Grunhagen et al. [41] reported that no significant difference in the CR-rate was found between patients receiving a melphalan-ILP with or without IFNγ (78% vs. 66%, respectively, *p* = 0.274). No study was able to establish clearly that the addition of TNF-α increased the rate of complete responses.

Two 12/24) studies compared the results of performing one ILP with repeated ILPs [25,27] (total *n* = 422 ILPs). In addition, Deroose et al. [26] and Grunhagen et al. [24] performed repeated ILPs and compared the results with those found in their center’s database based on just an initial ILP. None of them found statistically significant differences in the response rate after a first ILP or after repeated ILP.

Regarding temperature, all the studies were carried out in hyperthermia except those of Noorda, which also included ILPs carried out in normothermia. Only one study [29] analyzed the results reported separately using the data from using different temperatures at different durations (39–40 °C for 60 min; 39–40 °C for 90 min; 39–40 °C for 120 min or 41–41.5 °C for 120 min), finding a longer perfusion time (120 min) under mild hyperthermia (39–40 °C) as a predictive factor of CR.

Several studies analyzed the predictive factors of response. In the multivariate analysis, they found that the statistically significant predictive factors for the complete response were: a total number of metastases less than ten [27,29,33], a longer perfusion time (120 min) under mild hyperthermia (39–40 °C) [29], TNF dose [34,35], age < 65 years [34], and the absence of lymph node metastases [37,43] or at stage IIIB or less [34]. Disease stage was also a predictor of the complete response in the Deroose et al. [35]. No study was able to demonstrate that gender was included in the CR rate in multivariate analysis.

**Figure 2:**
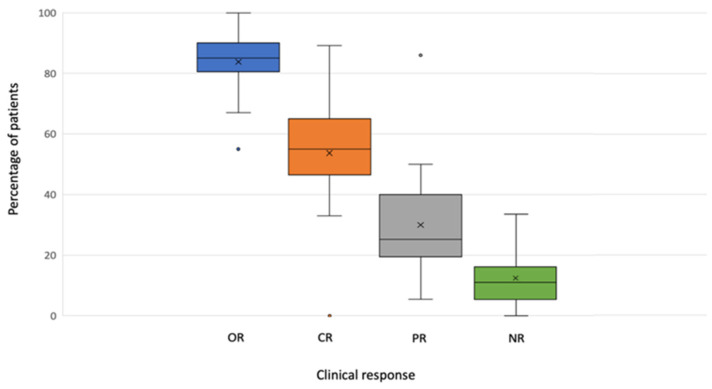
Boxplot of response by variables.

Data on clinical response are graphically represented in Figure 2.

### 3.2. Survival

Twenty-three studies (*n* = 2642) provided valid data on survival (Table 3). Of these, 17 studies (*n* = 2195) provided data on OS, reporting a median OS of 38 months (range: 17–56 months), as well as a median OS at 3, 5, and 10 years of 38%, 35%, and 16%, respectively.

Thirteen studies (*n*= 1060) [24,26,30,31,32,33,34,35,38,41,42,44] reported valid data on the rate of local progression-free survival (LPFS). A median of 56% (range: 46–63%) of the patients presented a local relapse with a median LPFS of 13 months (range: 6–17.4 months).

Regarding the impact on survival with the addition of TNF to melphalan therapy, six studies (*n* = 837) reported valid results [22,27,28,31,33,37]. None of them reported statistically significant differences between the treatment with melphalan + TNF vs. melphalan alone. The addition of TNF-α was also not identified as a predictive factor for survival in any study.

On the other hand, and considering the other drugs, Rossi et al. [22] included 31 patients in their pilot trial, comparing the combination with melphalan, TNF-α and IFN α-2b versus melphalan and TNF-α. A significant increase in PFS was demonstrated in the IFN α-2b group (median time to progression: 26 and 17 months, respectively; log-rank test *p*-value: 0.037). This survival benefit was confirmed by a multivariate analysis, where treatment was found to be an independent predictor of longer OS. Another parameter registered in five studies [30,31,33,35] was the melanoma-specific survival (MSS), with a median of 30 months (range 24–52 months).

The median time to local progression (TTLP) for patients with CR was 18 months (range: 10–23.8 months) [30,31,36,38,41,42], with a median of 5.7 months (range: 4–14 months) for patients with RP [30,31,37,41]. Both Madu et al., and Rossi et al., observed statistically significant differences for the LPFS of patients who presented with CR vs. those who did not (*p* < 0.001 and *p* < 0.0001, respectively). No study observed statistically significant differences in TTLP for the patients treated with melphalan alone vs. patients treated with melphalan plus TNF-α [31,34,37].

Five studies provided valid results on the time to systemic progression (TTSP) [24,36,38,39,40], with a median of 10.75 months (range: 7.5–19.7) for the general population. TTSP did not differ between patients receiving a single ILP (12 months) and multiple ILPs (15 months; *p* = 0.27) [24].

Belgrano et al. [27] and Grunhagen et al. [24] reported survival comparisons with an ILP and with repeated ILPs. Only Belgrano et al. (*n* = 380) reported differences in the overall survival, with a median OS of 34 months for the patients treated with one ILP, 41 months for patients treated with two ILPs, and 93 months for those who underwent three to five ILPs (*p* = 0.02). Grunhagen et al. [24] also did not observe significant differences in BPD after ILP, compared to re-ILP: the median time to local progression (TTLP) was 14 months for the repeated perfusion versus 16 months for the overall population and 18 months for single ILPs (*p* = 0.40).

Two studies compared the effectiveness of ILP based on the age of the patients: one of them stratified the patients at <75 and ≥75 years old [45] and another at ≤70 and >70 years old [30]. Noorda et al. [45]. found no significant differences in the rate of CR, recurrence, DFS, and OS between both groups. Madu et al. [30] reported a median MSS (45 months for patients ≤70 years of age, and 18 months for patients over 70 years of age) as the only difference found between the two groups (*p* = 0.038), showing that an age of over 70 years (*p* < 0.001, HR 3.86, 95% CI 1.94–7.71) increased the risk of death by melanoma.

Five studies [34,35,36,38,39] reported survival data stratified by the tumor stage. Stage IIIA (lymph node micrometastases in the previous AJCC staging system) was associated with a better survival in several studies. According to Deroose et al., 35 patients presenting with stage IIIA disease had 5- and 10-year, disease-specific survival rates of 47 and 31% with a median disease-specific survival of 58 months, compared with 12%, 4%, and 20 months in the stage IIIA-B group (*p* < 0.001). No patient with stage IV disease survived for more than 3 years.

The predictive survival factors were defined in 13 studies according to a multivariate analysis. The identified predictive factors of a higher survival were: a lower stage, smaller number of metastases, low Breslow index, an increased number of ILPs, CR, and a lower age. Additionally, Deroose et al. [34] identified sex as a prognostic factor for the time to systemic progression (TTSP). Alexander et al. [28] reported that the female sex was significantly and independently associated with prolonged in-field PFS, while only the female sex was shown to be associated with OS (*p* = 0.27). However, according to Rossi et al. [22], the only independent prognostic factor was treatment, with a risk reduction of 62% in favor of adding IFN to melphalan with a TNF-α therapy. Deroose et al. [34] analyzed BMI (body mass index) as a prognostic baseline factor, but did not reach a significant conclusion regarding the clinical outcome, nor for TTLP, TTSP, or OS.

### 3.3. Secondary Effectiveness Endpoints

The limb salvage rate (LSR) was analyzed in six studies, which included 474 patients in whom the only therapeutic alternative to ILP was amputation. They reported a median LSR of 94% with a median follow-up of 32 months (range 19–51 months).

### 3.4. Toxicity

#### 3.4.1. Locoregional Toxicity

All the studies included in this review, except for one [28], reported valid data on locoregional toxicity (*n* = 2546) (Table 4). Three of them [26,27,34] grouped mild toxicities (Wieberdink grade I and II) when reporting their results. A median of 44.4% of the patients (range 0–81%) did not present any toxicity (Wieberdink grade I). Grade II occurred in a median of 56% (range 30–83%); grade III in a median of 24.4% (range 0–38.2) and grade IV in a median of 2.5% (range 0–7%). A median of 0.2% of patients (range 0–3%) required amputation due to toxicity produced by ILP (grade V).

Two studies [21,43] reported valid data on locoregional toxicity comparing melphalan and melphalan plus TNF-α. Alexander et al. observed that the most significant systemic toxicities were associated with the use of TNF (transient hypotension was the most common). Cornett et al. [21] reported that grade 4 AEs were observed in 14 patients (11%), with 3 out of 64 patients (5%) in the melphalan-alone arm and 11 of 66 patients (17%) in the melphalan-plus-TNF arm (*p* = 0.028). However, Noorda et al. [43] found no difference in locoregional toxicity, complications or long-term morbidity. Rossi et al. [22] also did not observe a difference when adding IFN.

In relation to toxicity produced by other drugs, Papadia et al. [23] reported that the acute local toxicity of L19-TNF ILP was mild, most likely because TNF via L19-TNF was targeted directly to the tumor tissue using a much lower total TNF-α activity compared to TM-ILP. Rossi et al. [22] showed that a grade 2 toxicity was similar in the melphalan-plus-TNF group to that of the group with melphalan, TNF-α and IFN-γ (83% vs. 79%; *p* = 0.70).

Madu et al. [30] and Noorda et al. [45] observed that the incidence and severity of locoregional toxicity did not differ between age groups. Noorda et al. [45] did not find differences in the short- or long-term morbidity, or in other postoperative complications (seroma, local infection, etc.). Regarding the toxicities after the first ILP or after repeated ILP [25,26,27], no studies found significant differences (*p* = 0.664, *p* = 0.288, and *p* = 0.28, respectively). Katsarelias et al. [29] performed a multivariate analysis, comparing Wieberdink I–III versus IV–V and Wieberdink I-II versus III toxicities, and concluded that the perfusion at 41–41.5 °C for 120 min had a higher rate of severe toxicity (grade III–V), with an odds ratio of 3.9 (*p* = 0.04) and 2.59 (*p* = 0.05), respectively.

The predictive factors of toxicity were analyzed in four studies (*n* = 619) [21,29,33,40]. A more advanced age [33,40], longer perfusion time (120 vs. 90 min), higher perfusion temperature (41 °C vs. 40 °C) [29,33] and the female sex were identified as predictive factors of toxicity [40].

**Figure 3:**
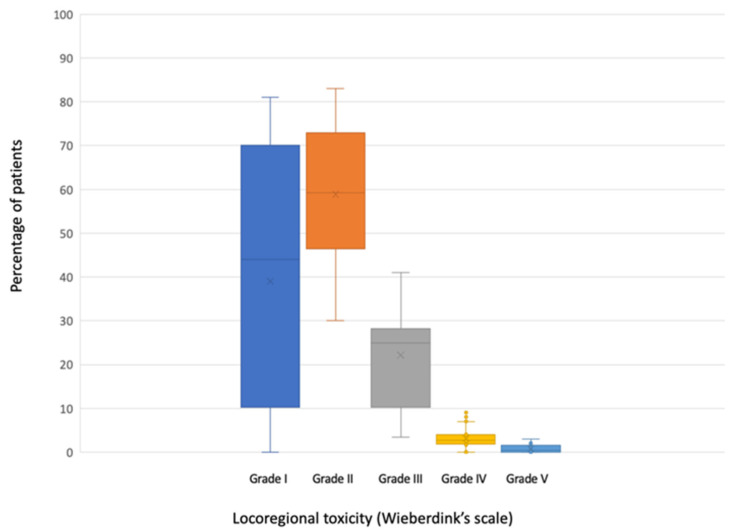
Boxplot of locoregional toxicity according to Wieberdink’s scale.

Data on locoregional toxicity are graphically represented in Figure 3.

#### 3.4.2. Systemic Toxicity

The systemic toxicity was reported in twelve studies [22,23,28,30,33,34,35,36,37,39,41] (Table 5) in a very heterogeneous manner; severe toxicities (myocardial ischemia, pulmonary embolism) were rare. The most commonly reported adverse effects were hematological (especially leukopenia, as well as thrombocytopenia and anemia), fatigue, fever, hypotension (this was transitory and treated with vasopressors, associated in some studies with TNF-α leaks), and the mild elevation of myoglobin. Four deaths were described at some point during hospital admission after ILP: three of a cardiac cause and one of a respiratory cause.

Finally, Belgrano et al. [27]. did not find differences in the systemic toxicity between the first and subsequent ILP (*p* = 0.54).

### 3.5. Complimentary Data

Seven studies reported the percentage of leakage measured during ILP [22,24,28,30,34,35,41]. The median was 0% (range 0–0%), with a median maximum leakage of 20.5 (range 7.4–32%).

Noorda et al. evaluated hospital stays in two studies [25,45]. In their 2016 study with re-ILP, they reported a median of 23 days (range 9–65 days), which was not significantly longer than after the first procedure (20 days, *p* > 0.05). In 2002 it was observed that patients older than 75 years of age stayed significantly longer in the hospital than younger patients. Additionally, the female sex, wound infection and more severe limb toxicity were risk factors for a longer hospital stay.

## 4. Discussion

In this review, we confirm that ILP still offers a high efficacy, with response results comparable to those reported in the review by Moreno-Ramirez et al. [46], with a median global response of 90% (64–100%) and median complete responses of 58% (25–89%). The most widely used and tested drugs are melphalan and TNF-α. Others, such as dacarbazine or dactonomycin, have been used in few centers and their efficacy is less well-established. Higher overall and complete response rates are obtained with the melphalan-TNF combination than with melphalan alone. However, it appears that the addition of TNF to ILP is associated with a greater toxicity and there is no survival benefit. In general, studies agree that the longer the time interval between the treatment of the primary tumor and the development of ITM and the lower the tumor burden, the better the MSS and overall survival. Thus, there is some consensus in most melanoma referral centers that the true indication for TM-ILP is the presence of a bulky disease or the event of relapse after previous M-ILP [35].

Performing more than one ILP for the same patient is safe and does not appear to increase locoregional toxicity. Even a higher number of re ILPs was described as a predictive factor for survival in the multivariate analysis performed by Belgrano et al. [27]. For recurrent metastases in transit, re-ILP still plays a role, especially if the patient responds after the first treatment.

ILP in the elderly offers response rates similar to those obtained in young patients and is safe, with no evidence of increased short-term morbidity or a higher incidence of postoperative complications.

Another factor described as a predictor of better survival was the treatment with IFN alpha 2b. In fact, in our center and others, interferon alfa 2b was administered to some patients in an effort to consolidate the antitumor effect of ILP, based on the results of the pilot study carried out by Rossi et al. [22] as justification. This clinical trial tested the hypothesis that the systemic administration of low-dose interferon alfa 2b could increase the duration of progression-free survival in patients undergoing TNF-based ILP. A statistically significant difference in progression-free survival of 26 vs. 17 months favoring the IFN group (P = 0.037) was observed. In addition, this survival benefit was confirmed at multivariate analysis, where treatment was the only prognostic factor retained by the prediction model and the analysis of the risk of disease progression over time suggested that this survival benefit appeared to vanish after IFN discontinuation.

At low doses, IFNα, the only drug that was used in adjuvant melanoma until the approval of targeted therapies and immunotherapy in recent years, appears to inhibit tumor angiogenesis by directly inhibiting endothelial cell proliferation and negatively regulating the expression of proangiogenic factors (e.g., VEGF, b-FGF, IL-8, and matrix metalloproteinases) [47].

**Figure 4:**
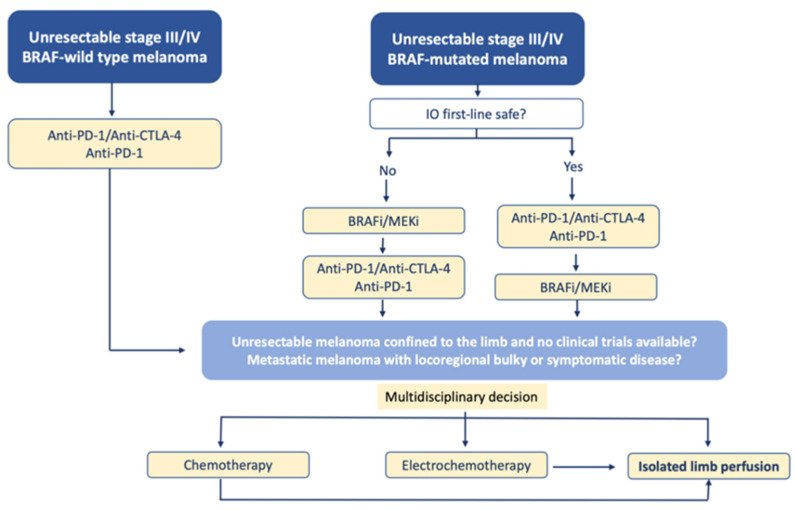
Proposal for a therapeutic algorithm for the management of patients with unresectable melanoma of the limbs including ILP (modified from ESMO guidelines).

Although the presence of disseminated disease was not a contraindication of ILP for in-transit melanoma, the current treatment of these patients is undoubtedly immunotherapy and targeted therapies, as shown by the following therapeutic algorithm that we propose (Figure 4):

Electrochemotherapy, as indicated in the previous algorithm, is a valid alternative to ILP in case of cutaneous and subcutaneous metastases of melanoma.

It is based on the phenomenon of reversible electroporation. In this method, by applying an electric current to the tissue, we induce a temporary increase in permeability of the cell membrane, thus enabling a free flow of large molecules into the cell, including cytostatics that, at baseline, are not transported to the cytosol. As a result, their potential toxicity increases considerably [48].

The studies published in relation to electrochemotherapy in melanoma, similar to the studies on ILP, are heterogeneous and generally include few patients. In a review of the literature carried out by Wichtowski [49], which included 12 publications, an OR of 74% (CR rate of 40.1% and PR rate of 34%), slightly lower than those described in our review was recorded, so this option could be chosen in centers where ILP is not available or when the patient is not a candidate for it.

The development of new systemic drugs in the last decade has radically changed the treatment of melanoma. The introduction of BRAF/MEK inhibitors and immune checkpoint inhibitors offers new hope for patients with stage IV melanoma. The specific response rates with these drugs for in-transit metastases have not been reported to date, but the results in terms of the overall survival are superior to those demonstrated in this review.

Immunotherapy studies report a 3-year OS rate from 50–58% and a 5-year OS rate from 34–44% with anti-PD1 as single therapies and 52% with the combination antiPD1 + anti CTLA 4 [50,51]. The targeted therapy studies show a median overall survival of 22.3 to 33.6 months [52,53]. Additionally, the updated results for dabrafenib-trametinib have recently been published [54] with a 5-year OS of 34%. It must be taken into account that the majority of patients included in these clinical trials had stage IV melanoma, while most of the patients in our review had stage III, that is, they presented an earlier stage of the disease.

Guadagni et al. [55] evaluated the current role of melphalan, hypoxic, pelvic perfusion in patients with advanced pelvic melanoma retrospectively. The overall median survival time (MST) stratified for variables, including the BRAF V600E mutation and eligibility for treatments with new immunotherapy drugs, was assessed in 41 patients with pelvic melanoma loco regional metastases who received a total of 175 treatments with melphalan hypoxic perfusion and cytoreductive excision. The first treatment resulted in a 97.5% response-rate in the full cohort and a 100% response-rate in the 22 wild-type BRAF patients. MST spent 18 months in the full sample, 20 months for the 22 wild-type BRAF patients and 21 months for the 11 wild-type BRAF patients not eligible for immunotherapy. Guadagni et al. conclude that Melphalan hypoxic perfusion is a potentially effective treatment for patients with locoregional metastases of pelvic melanoma and propose to determine if Melphalan pelvic perfusion under conditions of hypoxia may generate an immune response that could be augmented by systemic immunotherapy with anti-programmed cell death-ligand protein 1 (PD-L1) antibodies [56].

In this regard, the study by Ariyan CE et al. [57] combines isolated limb infusion (ILI) and ipilimumab and shows a positive synergistic effect. In this study, 26 patients with advanced melanoma were treated locally by ILI with the nitrogen mustard-alkylating agent melphalan, followed by the systemic administration of CTLA-4 blocking the antibody (ipilimumab) in a phase II trial. This combination of local chemotherapy with a systemic checkpoint blockade inhibitor resulted in a response rate of 85% at 3 months (62% complete and 23% partial response rate) and a 58% progression-free survival at 1 year. The clinical response was associated with an increased T-cell infiltration, similar to that seen in the murine models. All together, these findings suggest that local chemotherapy combined with checkpoint blockade-based immunotherapy may synergize and induce a durable response to cancer therapy.

It would also be interesting to analyze the role of ILP in some subtypes of melanomas with a worse response to immunotherapy (such as acral or mucosal melanomas). Although there are also no clinical trials in this setting, their role would be similar to that of other melanomas, that is, it could be assessed as a palliative treatment after the progression to immunotherapy or targeted therapies.

Finally, a major limitation of our review is the age and the great heterogeneity between the included studies (most of them with a small number of patients and carried out retrospectively), which makes it difficult to transfer their results to the current era.

## 5. Conclusions

ILP, with its low incidence of regional and systemic toxicity, is a valuable palliative treatment not only for patients with a disease confined to the limbs, but also for patients with metastatic melanoma with bulky or symptomatic diseases to improve their quality of life. Therefore, we believe that this procedure should still be considered when the rest of the highly effective systemic therapies available at the present time have failed, especially in cases where local disease morbidity (ulceration, painful and bleeding lesions, and others) is a major challenge, and other locoregional strategies, such as electrochemotherapy are not indicated or are ineffective. Above all, ILP must always be considered in cases where amputation could eventually be indicated.

The clinical trials that combine ILP, intralesional and systemic therapies are underway and the first preliminary results seem encouraging. Hopefully, the emerging new data from these combinatorial strategies could clarify the future role of ILP in the global management of locoregional melanoma disease.

## Figures and Tables

**Table 1:** Studies of ILP for unresectable locally advanced melanoma of the limbs included in the systematic review.

Study	Country	*n* of ILPs Included	Median Age (yr)	Study Design	Chemotherapy Regimen	Outcomes Evaluated
Belgrano et al. (2019) [27]	Sweden	380	68	R	Repeated Mel with or without TNF	Effectiveness Toxicity
Katsarelias et al. (2018) [29]	Sweden	284	70.5	R	Mel	Effectiveness Toxicity
Madu et al. (2017) [30]	The Netherlands	91	70	R	Mel with or without TNF	Effectiveness Toxicity
Deroose et al. (2015) [26]	France	37	63	R	Repeated Mel + TNF	Effectiveness Toxicity
Hoekstra et al. (2014) [31]	The Netherlands	60	65	R	Mel with or without TNF	Effectiveness Toxicity
Paulsen et al. (2014) [32]	Denmark	84	63	R	Mel with or without TNF	Effectiveness Toxicity
Olofsson et al. (2013) [33]	Sweden	163	70	R	Mel with or without TNF	Effectiveness Toxicity
Papadia et al. (2013) [23]	Italy	17	65	CT	Mel + L19-TNF	Effectiveness Toxicity
Deroose et al. (2012) [34]	The Netherlands	167	65	P	Mel + TNF	Effectiveness Toxicity
Deroose et al. (2011) [35]	The Netherlands	118	64	P	Mel + TNF	Effectiveness Toxicity
Pace et al. (2011) [36]	Italy	91	61.6	P	Mel + Dactin	Effectiveness Toxicity
Rossi et al. (2010) [37]	Italy	112	62.1	R	Mel with or without TNF	Effectiveness Toxicity
Boesch et al. (2010) [38]	Germany	152	68	P	Mel + Dactin	Effectiveness Toxicity
Alexander et al. (2009) [28]	USA	91	57	P	Mel with or without TNF (+IFNγ)	Effectiveness
Rossi et al. (2008) [22]	Italy	12 19	63 61	CT	Mel + TNF + INF α-2b Mel + TNF	Effectiveness Toxicity
Knorr et al. (2006) [39]	Germany	100	52	R	Mel + Dacarb	Effectiveness Toxicity
Cornett et al. (2006) [21]	USA	65 68	60 66	CT	Mel Mel + TNF	Effectiveness Toxicity
Noorda et al. (2006) [25]	The Netherlands	42	65	R	Mel with or without TNF (+IFNγ) Repeated Mel + TNF (+ IFNγ)	Effectiveness Toxicity
Grunhagen et al. (2005) [24]	The Netherlands	25	60	R	Repeated Mel + TNF (+ IFNγ)	Effectiveness Toxicity
Aloia et al. (2005) [40]	USA	59		R	Mel	Effectiveness
Grünhagen et al. (2004) [41]	The Netherlands	100	62	R	Mel + TNF (+IFNγ)	Effectiveness Toxicity
Rossi et al. (2004) [42]	Italy	20	63	R	Mel + TNF	Effectiveness Toxicity
Noorda et al. (2004) [43]	The Netherlands	130	67	R	Mel with or without TNF (+IFNγ)	Effectiveness Toxicity
Noorda et al. (2004) [44]	The Netherlands	43	62	R	Mel with or without TNF (+IFNγ)	Effectiveness Toxicity
Noorda et al. (2002) [45]	The Netherlands	57 158	79 60	R	Mel with or without TNF ≥ 75 year-old Mel with or without TNF < 75 year-old	Effectiveness Toxicity

Abbreviations: CC, case–control study; CS, case series; Dacarb, dacarbazine; Dactin: Dactinomycin; ILP, isolated limb perfusion; Mel, melphalan; P, prospective; R, retrospective; CT, clinical trial; TNF, tumoral necrosis factor-α; IFN, interferon.

**Table 2:** Clinical response to ILP in studies included in the systematic review.

Study	Clinical Setting	ILP Regimen	T	*n* ILPs	OR (%)	CR (%)	PR (%)	NR (%)	LSR (%)
Belgrano et al. (2019) [27]	ULAM	Repeated Mel with or without TNF	H	380	81.00	47.00			
		Mel		263	85.00	63.00			
	1st ILP	Mel + TNF		27	67.00	33.00			
		Mel		17	55.00	36.00			
	Re-ILP	Mel + TNF		73	81.00	47.00			
Katsarelias et al. (2018) [29]	ULAM	Mel	H	268	83.20	58.80	24.40	16.80	
Madu et al. (2017) [30]	ULAM ≤70 year-old >70 year-old	Mel with or without TNF	N	91 44 47	81.00 83.00 80.00	47.00 45.00 48.00	34.00	19.00	96.70
Deroose et al. (2015) [26]	ULAM	Repeated Mel + TNF	H	37	86.00	65.00	21.00	14.00	
Hoekstra et al. (2014) [31]	ULAM	Mel with or without TNF Mel Mel + TNF	H	60 19 41	90.00 84.00 93.00	45.00 33.00 54.00	45.00	5.00	93.00
Paulsen et al. (2014) [32]	ULAM	Mel with or without TNF	H	84	85.00	42.00	43.00	15.00	
Olofsson et al. (2013) [33]	ULAM	Mel with or without TNF	H	155	85.00	65.00	20.00	15.00	
Papadia et al. (2013) [23]	ULAM	Mel + L19-TNF 325 µg Mel + L19-TNF 650 µg	H	7 10	86.00 89.00	0.00 50.00	86.00 39.00	14.00 11.00	
Deroose et al. (2012) [34]	ULAM	Mel + TNF Mel + TNF 3–4 mg Mel + TNF 1–2 mg	H	167	89.00	61.00 70.00 49.00	28.00	11.00	
Deroose et al. (2011) [35]	ULAM	Mel + TNF	H	118	93.20	67.80	25.40	6.80	92.40
Pace et al. (2011) [36]	ULAM	Mel + Dactin	H	56	94.60	89.20	5.40	5.40	
Rossi et al. (2010) [37]	ULAM	Mel with or without TNF	H		90.10	51.40	38.70	9.90	
		Mel		53	90.60	41.50	49.10	9.40	
		Mel + TNF		59	89.60	60.30	29.30	10.30	
Boesch et al. (2010) [38]	ULAM	Mel + Dactin	H	145	80.70	62.80	17.90	19.30	
Alexander et al. (2009) [28]	ULAM	Mel with or without TNF (+ IFNγ)	H	90	95.00	69.00	26.00		
Rossi et al. (2008) [22]	ULAM	Mel + TNF (+ IFN α-2b sc) Mel + TNF	H H	12 19	100.00 100.00	50.00 53.00	50.00 47.00	0.00 0.00	
Knorr et al. (2006) [39]	ULAM IV			100					
	IIIA MD	Mel + Dacarb	H	40	80.00	65.00	15.00	2.00	
	IIIAB MD	Mel + Dacarb	H	51	80.00	55.00	25.00	8.00	
	IV MD	Mel + Dacarb	H	9	67.00	45.00	22.00	33.00	
Cornett et al. (2006) [21]	ULAM	Mel Mel + TNF	HH	58 58	79.00 95.00	64.00 69.00	25.00 26.00	39.00 43.00	
Noorda et al. (2006) [25]	ULAM	Mel with or without TNF Repeated Mel + TNF	N/H	17 21	77.00 72.00	65.00 62.00	12.00 10.00	18.00 5.00	95.00
Grunhagen et al. (2005) [24]	ULAM	Repeated Mel + TNF (+ IFNγ)	H	25	96.00	76.00	20.00	4.00	
Aloia et al. (2005) [40]	ULAM	Mel	H	58	88.00	57.00	31.00	12.00	
Grünhagen et al. (2004) [41]	ULAM	Mel + TNF (+ IFNγ)	H	100	95.00	69.00	26.00	5.00	
Rossi et al. (2004) [42]	ULAM Bulky disease	Mel + TNF	H	20	95.00	70.00	25.00	5.00	
Noorda et al. (2004) [43]	ULAM	Mel Mel + TNF (+ IFNγ)	N H	40 90		45.00 59.00			96.00
Noorda et al. (2004) [44]	ULAM	Mel with or without TNF (+IFNγ)	H/N	43	84.00	64.00	20.00	4.00	93.00
Noorda et al. (2002) [45]	ULAM ≥ 75 year-old ULAM < 75 year-old	Mel with or without TNF Mel with or without TNF	H/N H/N	57 158		56.10 58.20			

Abbreviations: CR, complete response; Dacarb, dacarbazine; Dactin, Dactinomycin; H, hyperthermia; IFN, interferon; ILP, isolated limb perfusion; LSR, limb salvage rate; MD, MD Anderson staging classification system for malignant melanoma; Mel, melphalan; N, normothermia; NR, no response; OR, overall response; PR, partial response; T, temperature regimen; TNF, tumor necrosis factor; ULAM, unresectable locally advanced melanoma.

**Table 3:** Survival results of ILP for unresectable locally advanced melanoma.

Study	Clinical Setting	ILP Regimen	T	*n* of ILPs	5-yr OS (%)	3-yr OS (%)	Median OS Interval (mos)	Median MSS (mos)	Median PFS Interval (mos)
Belgrano et al. (2019) [27]	ULAM	Repeated Mel with or without TNF 1st ILP 2nd ILP 3–5th ILP	H	380 290 68 22	37.00		39.00 34.00 41.00 93.00		
Katsarelias et al. (2018) [29]	ULAM	Mel	H	284	36.00		38.00		
Madu et al. (2017) [30]	ULAM ≤70 year-old >70 year-old	Mel with or without TNF	N	91				38.00 45.00 18.00	6.00
Deroose et al. (2015) [26]	ULAM	Repeated Mel + TNF	H	37	35.00	56.00	45.00		17.00
Hoekstra et al. (2014) [31]	ULAM		H					52.00	
		Mel		19				51.00	
		Mel + TNF		41				68.00	
Paulsen et al. (2014) [32]	ULAM	Mel with or without TNF	H	84	31.00		17.00		7.00
Olofsson et al. (2013) [33]	ULAM	Mel with or without TNF	H	155	26.00		27.00	30.00	
Deroose et al. (2012) [34]	ULAM	Mel + TNF	H	167	26.00	40.00		27.30	
		High dose							16.00
		Low dose							11.00
Deroose et al. (2011) [35]	ULAM	Mel + TNF	H	118				24.00	
Pace et al. (2011) [36]	ULAM	Mel + Dactin	H	91	45.00		37.00		
Rossi et al. (2010) [37]	ULAM	Mel with or without TNF Mel Mel + TNF	H	112	28.50		34.80 33.50 34.80		
Boesch et al. (2010) [38]	ULAM	Mel + Dactin	H	152	34.00		39.00		
Alexander et al. (2009) [28]	ULAM	Mel with or without TNF (+IFNγ)	H	90	43.00		47.40		12.40
Rossi et al. (2008) [22]	ULAM	Mel + TNF (+INF α-2b sc) Mel + TNF	H H	12 19					26.00 17.00
Knorr et al. (2006) [39]	ULAM						42.00		21.00
	IIIA MD	Mel + Dac	H	40	47.00				
	IIIAB MD	Mel + Dac	H	51	35.00				
	IV MD	Mel + Dac	H	9	34.00				
Grünhagen et al. (2005) [24]	ULAM	Repeated Mel + TNF (+IFNγ)	H	25					
		Single ILP		4	28.00				
		Multiple ILP		21	47.00				
Aloia et al. (2005) [40]	ULAM	Mel	H	58		54.00			13.40
Grünhagen et al. (2004) [41]	ULAM	Mel + TNF (+IFNγ)	H	100	32.00		25.00		
Noorda et al. (2004) [43]	ULAM	Mel Mel + TNF	NH	40 90	29.00				
Noorda et al. (2004) [44]	ULAM	Mel with or without TNF	H/N	43	46.00		56.00		
Noorda et al. (2002) [45]	ULAM ≥ 75 year-old ULAM < 75 y-old	Mel with or without TNF Mel with or without TNF	H/N H/N	57 158	40.60 37.00				

Abbreviations: Dacarb, dacarbazine; Dactin, Dactinomycin; DFS, disease-free survival; H, hyperthermia; ILP, isolated limb perfusion; MD, MD Anderson staging classification system for malignant melanoma; Mel, melphalan; MSS, melanoma specific survival; N, normothermia; OS, overall survival; T, temperature regimen; TNF, tumor necrosis factor; ULAM, unresectable locally advanced melanoma.

**Table 4:** Regional toxicity of ILP.

Study	ILP Regimen	*n* ILPs	Wieberdink Grade ^a^ (%)				
			I	II	III	IV	V
Belgrano et al. (2019) [27]	Repeated Mel with or without TNF 1st ILP 2nd ILP 3rd–5th ILP	308	63.00 70.00 67.00 59.00		30.00 27.00 24.00 41.00	7.00 3.00 9.00 0.00	
Katsarelias et al. (2018) [29]	Mel + TNF (+ IFNγ)	270	4.40	62.60	24.40	8.10	0.40
Madu et al. (2017) [30]	Mel with or without TNF	91				2.20	0.00
Deroose et al. (2015) [26]	Repeated Mel + TNF	37				2.00	
	1 ILP		22.00	54.00	21.00		1.00
	Re-ILP		70.00		27.00		2.70
Hoekstra et al. (2014) [31]	Mel with or without TNF	60		63.00	28.00	7.00	2.00
Paulsen et al. (2014) [32]	Mel with or without TNF	84	44.00	43.00	11.00	3.00	
Olofsson et al. (2013) [33]	Mel with or without TNF	161	0.00	63.00	33.00	3.00	0.00
Papadia et al. (2013) [23]	Mel + L19-TNF						
	Mel + L19-TNF 325 µg	7	0.00	71.40	28.60	0.00	0.00
	Mel + L19-TNF 650 µg	10	60.00	40.00	0.00	0.00	0.00
Deroose et al. (2012) [34]	Mel + TNF	167	18.00	56.00	23.00	2.00	1.50
Deroose et al. (2011) [35]	Mel + TNF	118	71.20		25.40	2.50	1.69
Pace et al. (2011) [36]	Mel + Dactin	91	5.40	54.30	38.20	2.10	0.00
Rossi et al. (2010) [37]	Mel Mel + TNF	53 59	15.10 45.80	77.40 47.50	7.50 3.40	0.00 1.70	0.00 1.70
Boesch et al. (2010) [38]	Mel + Dactin	152			8.00	4.00	1.00
Rossi et al. (2008) [22]	Mel + TNF (+ INF α-2b sc) TNF	12 19		83.00 79.00			
Knorr et al. (2006) [39]	Mel-Dac	100			6.00	4.00	1.00
Cornett et al. (2006) [21]	Mel Mel + TNF	64 66				2.00 3.00	0.00 3.00
Grünhagen et al. (2004) [41]	Mel + TNF (+ IFNγ)	100	15.00	54.00	27.00	3.00	1.00
Rossi et al. (2004) [42]	Mel + TNF	20	65.00	30.00	5.00	0.00	0.00
Noorda et al. (2004) [43]	Mel Mel + TNF	40 90	71.00 75.00		26.00 23.00	3.00 2.00	
Noorda et al. (2004) [44]	Mel with or without TNF	43	69.00		28.00	2.63	0.00
Noorda et al. (2002) [45]	Mel with or without TNF (≥75 year-old) Mel with or without TNF (<75 year-old)	57 158	81.00 72.10		19.00 27.90		0.00 0.00

Abbreviations: Dacarb, dacarbazine; Dactin, Dactinomycin; H, hyperthermia; IFN, interferon: ILP, isolated limb perfusion; MD, Anderson staging classification system for malignant melanoma; Mel, melphalan; N, normothermia; T, temperature regimen; TNF, tumor necrosis factor; ULAM, unresectable locally advanced melanoma. ^a^ Wieberdink grade for limb toxicity: Grade I: No subjective or objective evidence of reaction; Grade II: Slight erythema and/or edema; Grade III: Considerable erythema and/or edema with some blistering, slightly disturbed motility; Grade IV: extensive epidermolysis and/or obvious damage to the deep tissue causing definite functional disturbances, threatening or manifest compartment syndrome; Grade V: Severe reaction which may necessitate amputation.

**Table 5:** Systemic toxicity of ILP.

Study	Clinical Setting	ILP Regimen	*n* ILPs	Blood		Gastrointestinal		Kidney		Respiratory		Cardiovascular		Neurologic	
				III	IV	III	IV	III	IV	III	IV	III	IV	III	IV
Papadia et al. (2013) [23]	ULAM	Mel + L19-TNF 325 µg Mel + L19-TNF 650 µg	7 10	28.57 60.00	14.28 30.00										
Rossi et al. (2008) [22]	ULAM	Mel + TNF TNF	12 19												
Knorr et al. (2006) [39]	ULAM	Mel-Dac	100												
Cornett et al. (2006) [21]	ULAM	Mel Mel + TNF	58 58	6.00 6.00						0.00 5.00		8.00 12.00			
Grünhagen et al. (2004) [41]	ULAM	Mel + TNF	100	0.00	1.00	0.00	0.00	0.00	0.00						
Noorda et al. (2004) [43]	ULAM	Mel Mel + TNF	40 90											4.00 2.00	

Abbreviations: III, IV, World Health Organization classification grade III and grade IV toxicitu; ILP, isolated limb perfusion; Mel, melphalan; N, normothermia; T, temperature regimen; TNF, tumor necrosis factor.
